# A Study and Development of Workplace Facilities and Working Environment to Increase the Work Efficiency of Persons with Disabilities: A Case Study of Major Retail and Wholesale Companies in Bangkok

**DOI:** 10.1155/2018/3142010

**Published:** 2018-08-19

**Authors:** Soraj Pruettikomon, Chaturong Louhapensang

**Affiliations:** Faculty of Industrial Education, King Mongkut's Institute of Technology Ladkrabang, Chalongkrung Rd. Ladkrabang, 10520, Bangkok, Thailand

## Abstract

This research aims to improve the work effectiveness of people with physical disabilities in department stores, retail and wholesale companies in Bangkok. It focuses on the environment and facilities needed by people with three types of disabilities, visual impairment, hearing impairment, and wheelchair users. A mixed research method was used (questionnaires, interviews, and observations). The results were applied to a design process based on the needs of people with physical disabilities and addressed solving their work problems, increasing their work effectiveness, and bringing the results of the design to a group discussion. The group was made up of 15 experts who were supervisors, representatives of the people with each type of physical disability, instructors, and personnel from the university knowledgeable about universal design for people with disabilities. Architects and designers helped to brainstorm ideas and develop designs that led to production and product testing. Testing was conducted to compare pretest and posttest results using designed products to evaluate the work effectiveness of the people with physical disabilities using statistical analysis (paired t-tests). The results using developed products showed a higher average posttest score than pretest. This indicated a statistically higher work effectiveness (*α* =0.05) and supports the research hypothesis.

## 1. Introduction

Based on a survey, it was found that the proportion of people with physical disabilities in Thailand is two percent, or two out of 100 people. There are around 2 million people with disabilities in Thailand. People with physical disabilities are characterized by visual and hearing impairments. Others are wheelchair users or have behavioral, mental, intellectual, or learning disabilities. In total, there are 31 impairments classified as physical disabilities. Moreover, the demographics of the Thai population has changed due to reduced birth rates and increased longevity. Thus, Thai society has become an aging society. Consequently, the elderly are at risk of developing disabilities and the frequency of disabled persons will rapidly increase. Most of the Thailand's disabled persons cannot support themselves and are considered a burden to society. The most severe problem is that people with physical disabilities do not have secure jobs. There are relatively few disabled workers employed in businesses [1].

The researchers analyzed the employment people with physical disabilities and summarized their greatest problems.These are education, work environment, and ease of workplace accessibility, as well as encouragement of the disabled. Without efforts to solve these problems, they will be exacerbated and the disabled will have great difficulties. If the number of people with physical disabilities increases, their problems could increase to a degree that able-bodied people give up trying to help them. The researchers studying employment of the disabled under the topic “career promotion guidelines of the disabled” suggested that the fundamental issues that are important to the disabled in Thailand are discrimination and opportunities for employment. Although the government has attempted to promote occupational training for the disabled, its efforts were not correctly focused and have produced no tangible results [3].

To improve the quality of life of the disabled, it is absolutely necessary to concurrently develop in various areas, as well as gain cooperation from many stakeholders, including members of society, private concerns, and public participants. According to one study [4], there are four fundamental disability rehabilitation systems, occupational, education, medical, and societal. Additionally, support tools for rehabilitation must be created. These include workplace facilities and work environments, provision for benefits, education to improve the attitudes of the disabled and society, and removal of obstacles. According to the Department of Empowerment of Persons with Disabilities, Ministry of Social Development and Human Security [1], there is a need for integration of the disabled into society by granting access and equal entitlement. There must not be any discrimination against people with disabilities. Moreover, good environments should be created, as well as equal access to technology and information for the disabled. Entitlements of people with disabilities from the government include subsidies such as annual payments received from the fund for Empowerment of Persons with Disabilities and mandated access to work. The desired improvement in the quality of life of the disabled was not realized. Companies are willing to pay into the disability rights fund. Only ~60,000 disabled people have been recruited into the system, although the rate has fluctuated. Of the two million disabled persons in Thailand, only 32,573 have applied to the system. Less than 3% of all employed people have physical disabilities. Many companies do not employ the disabled. So, they pay into the fund for Empowerment of Persons with Disabilities annually according to the Empowerment of Persons with Disabilities Act, B.E. (2007), Sections 33, 34, and 35. However, this does not necessarily improve the plight of the disabled.

Some studies showed that the solutions to these problems should be supported by legislation to enable access in society to the disabled, by the Ministry of Labour. Additionally, the Ministry of Social Development and Human Security should organize educational programs among various agencies to develop occupational training, instructional media, and skills development for the disabled. A fund was set up so that people with disabilities have the means to help themselves by improving the convenience and speed of the social system, as well as by decentralizing administration and improving their workplace environment. The laws are not applied to the public sector but are enforced in the private sector. According to the Department of Empowerment of Persons with Disabilities [1], the guidelines for compliance are as follows: Any employer or owner of a business must recruit or employ a least 1 disabled person for every 100 employees. If the number of a company's employees does not exceed 50 people, at least one disable person must be employed, as stipulated in the Empowerment of Persons with Disabilities Act, B.E. (2007) Section 33. If the employer or owner of business cannot comply with Section 33, they are obligated to contribute to the Fund for Promotion and Development of Life Quality of Disabled Persons or provide a location where goods or services are sold, hire a contractor to provide internships for persons with disabilities, or provide other assistance to the disabled or their caregivers, in accordance with the Empowerment of Persons with Disabilities Act, B.E. (2007), Section 35. Currently, employment of people with physical disabilities is done nationwide. The area with the highest employment of people with disabilities is metropolitan Bangkok. Moreover, Bangkok offers transportation services such as buses, the BTS, and taxis, among other modes. It is easier to travel here than in the provinces. From a survey of the employment of disabled persons in B.E. 2558 (2015) by the Department of Empowerment of Persons with Disabilities, 5,370 companies in Bangkok must employ disabled persons in accordance with the Empowerment of Persons with Disabilities Act, B.E. (2007), Section 33. In this city, there are 12,455 people between the ages of 15-70 years with physical disabilities. The highest proportion of these people have mobility or physical disabilities, followed by hearing impairment. There are 10,625 such people working in major retail and wholesale companies in Bangkok such as Makro, Big C, and Lotus. The disabled are employed for general work, administration, cleaning, stocking, and warehouse staff. From this information, it can be seen that people with disabilities can pursue careers and develop their capabilities if opportunities and support are provided in good work environments with adequate facilities. Safe work areas, signs for the hearing impaired, and convenient transportation as well as training to educate people with disabilities and their colleagues are needed [1].

In Thai society, people with physical disabilities are seen as a burden, creating problems and needing help. People who help the disabled are considered doing good deeds. The disabled often wait for help and do not want to rely on themselves. Consequently, this creates opportunities for activities in which they exploit the sympathies of the public using their disabilities. Occupational development of disabled people in Thailand failed and is seen as problematic. Despite the efforts and budget provided by the public and private sectors, concrete results have not been realized. The problems of employing the disabled may affect the way of life of ordinary people in society. As a result, the researchers are interested in finding guidelines to create workplace environments and facilities to properly and suitably assist the disabled. The researchers of the current study examined people with disabilities in three major retail companies, Makro, Big C, and Lotus. Their aims were to gather the results of the study and make improvements in work environments and workplace facilities for the disabled. Focus has been placed on having disabled people rely on themselves so that they can live in society. They need to increase their work effectiveness, generate income, enhance the quality of life for themselves and their families, and provide an example for people with disabilities in other occupations [5].

### 1.1. Research Objectives

The current study has three objectives:

(1) To study the theories, issues of workplace facilities, and environments in existing offices and use the resulting information to develop guidelines for large retailers and wholesalers in Bangkok.

(2) To design workplace facilities and environments for people with disabilities to reduce problems and work barriers and increase work effectiveness in department stores, retail and wholesale companies in Bangkok, using the principles of universal design.

(3) To test the effectiveness of the design of workplace facilities and environments for people with disabilities in department stores, retail and wholesale companies in Bangkok, using statistical principles and summarize the findings.

The products designed in the course of this study were various types of office furniture and cabinets to aid the disabled.

## 2. Literature Review

This research aims to collect data for design and development to increase work effectiveness for people with physical disabilities [6] in department stores, retailers, and wholesalers in Bangkok. The researchers used the following conceptual framework:

(1) The theory of creative equality on career and society: social enterprise is presented as a new alternative for people with disabilities to improve their quality of life thereby increasing their income and well-being. It is a shift in societal attitudes from the traditional perspective of seeing the disabled as a burden to becoming social assets. Having a disability does not mean being a burden. Social enterprises for people with disabilities help us learn about their capacities and values. It is a tool that empowers the disabled, providing training and occupational guidance. Therefore, people with physical disabilities can proudly rely on themselves, become empowered to live independently, have access to various services, and become equal members of society. This will eliminate discrimination, promote acceptance, and lead to a happier society [3].

(2) The theory of universal design refers to the design of the environment, workplace, and facilities so that all people in society can equally obtain benefits. The principle of universal design has 14 components: (1) Equitability, (2) Flexibility, (3) Simplicity, (4) Perceptible Information (i.e., information that can be understood by its users), (5) Tolerance of Error, (6) Low Physical Effort, (7) Size and Space, (8) Accessibility (i.e., users can get help using a device), (9) Access (i.e., users can take advantage of a device without assistance after training), (10) Ease of Reach, (11) Usability, (12) Safety, (13) Workability, and (14) Barrier-free or Non-handicapping designs [1].

## 3. Methods

The research process was divided into three phases. Mixed research methods were used [5]. Each phase is as follows:

**Phase 1 .**

Data was collected from department stores, retailers, and wholesale companies in Bangkok. The study consulted people with three types of disability and their trainers. Information was gathered about the work of people with physical disabilities as well as the needs and problems of employing of the disabled. The data were collected using questionnaires, observations, and interviews [7]. The sample consisted of 120 participants who worked in the three major retail and wholesale companies. The participants were 30 physically disabled people, 30 who were visually impaired, 30 with hearing impairment, and 30 trainers of the disabled to identify problems, obstacles, and needs. Thus, solutions to problems were identified and used in product design [8]. These designs were based on the information and needs of these disabled people and their trainers.

**Phase 2 .**

Discussions were held to summarize the information. The participants included experts on these types of disabilities and universal design, colleagues of the disabled, and the disabled themselves working in major retail and wholesale companies. This was done to brainstorm ideas on design and development of a draft summarizing the results of Phase 1. A discussion group was formed consisting of experts from department stores, retail and wholesale companies, instructors and design experts on universal design, and experts on these three types of disabilities. A total of 15 participants brainstormed for appropriate designs and ideas for development of products. The results of this session were used to develop the research products.

**Phase 3 .**

The products were brought to production and testing with the disabled in three major department stores, retail and wholesale companies. The test was administered before and after use of the office furniture designed in the current study. This was done to evaluate the effectiveness of the design with a sample of ten disabled people and do analysis using a paired t-test [9].

## 4. Results

**Phase 1 .**

The researcher summarized the data based on three types of disabilities and the opinions of close companions, i.e., their trainers. There were 120 participants who worked in three major department stores, retail and wholesale companies. The participants consisted of 30 physically disabled people, 30 who were visually impaired, 30 with hearing impairment, and 30 trainers. They identified problems, barriers, and needs to obtain solutions [6], as indicated in Table 1.

Table 1 shows a summary of quantitative data collected from questionnaires. The data will be collected first, followed by qualitative data collection by employing interviews and observations. This refers to sequential explanatory design [8].

**Figure 1:**
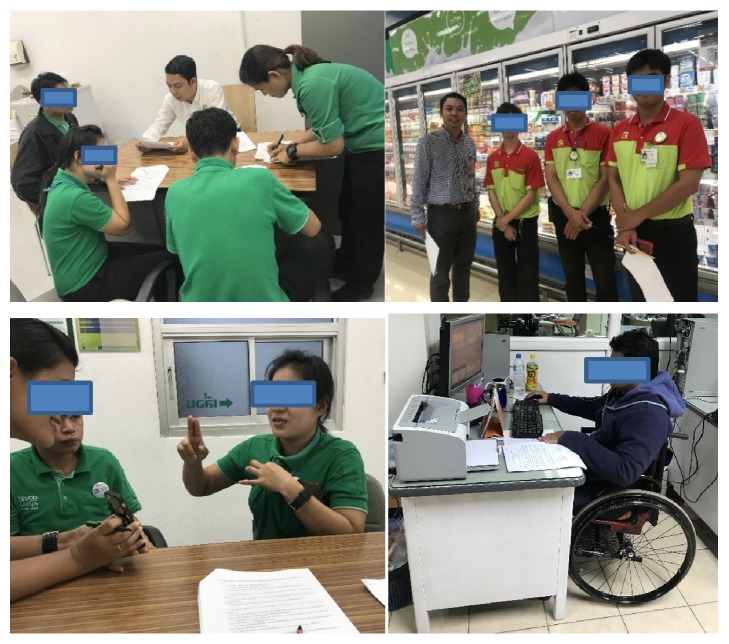
The researcher collected data in Phase 1 using questionnaires, observations, and interviews with three groups of disabled people in major retail and wholesale companies in Bangkok. The trainer interviews were conducted and data was collected from 120 questionnaires [2]. Source: the current study.

A summary of interviews and observations of the hearing impaired revealed that the disabled come to work on foot or by bus (see Figure 1: the researcher collected data in Phase 1 using questionnaires, observations, and interviews with three groups of disabled people in major retail and wholesale companies in Bangkok). The distance between their homes and work is not very far. Moreover, they do not prefer hard and difficult work as they do not wish to make decisions concerning their jobs. Most who submitted their resignations from work did so due to a lack of understanding of their coworkers, lower salary increases than colleagues who are not disabled, and fewer opportunities for job growth. The disabled have difficulty understanding their supervisors' instructions, exchanging information with their peers, following up on work orders, and negotiating with customers to fix work situations. Disabled people often sulked and were misunderstood, but they are more attentive to their work than others. The hearing impaired wanted to have a device to help them communicate and give instructions. The device must be lightweight and portable. Moreover, they want a fire alarm or warning system. Everyone likes to work in their chosen position in selected companies rather than working freelance due to the instability of income when working in a nontraditional position. Hearing impaired individuals want to work because they have to take care of their families and save money for the retirement. The hearing impaired think that a disability is not a problem in the workplace. However, they may feel discouraged due to communication issues and have few friends due to their hearing impairment. The most problematic issue in the workplace is placement of goods because they must follow instructions of their supervisors. They can communicate using the LINE application or through written instructions. However, in the event of confusion, they cannot seek clarification from their coworkers. Such barriers to work prevent the disabled from performing tasks due to problems in communications with colleagues or supervisors.

Environmental issues tend to be physical barriers in the workplace. These problems are related to signs and inability to hear alarms as well as difficulties in reading and understanding instructions and warnings, e.g., cautions about lifting heavy loads and instructions about using lifting or towing equipment. There were five recorded accidents in the workplace. For example, a person was nearly hit by a forklift because he did not hear aural warnings to get out its way. Another fell down a staircase and there were incidents where objects fell when disabled people placed materials incorrectly as a result of not understanding instructions. Communication needs to be improved. For example, having people who can understand sign language or use devices to help people with hearing impairments will help. Use of price checking machines is problematic. The hearing impaired cannot ask questions or understand how to use them because their difficulties prevent communication with colleagues and supervisors. Thus, they have to learn about the machine themselves. People with hearing disabilities think that communication aids will help them communicate and work more effectively.

A summary of interviews and observations revealed that people with physical disabilities are very attentive to work (see Figure 1: the researcher collected data in Phase 1 using questionnaires, observations, and interviews with three groups of disabled people in major retail and wholesale companies in Bangkok). However, they tend want to negotiate their working and quitting times to a greater degree than others. They live in close proximity to their workplace and prefer to work in a department where the physical workload is not very heavy. They tend to have a lower to upper secondary school education. People with physical disabilities do not like decision-making. They enjoy service work and follow instructions well after they understand what is needed. Most who resign from their jobs do so as a result of a lack of understanding between the c-workers, lower salary increases than other workers, and few opportunities for job growth. Their basic salary is lower than regular employees. Many people with physical disabilities sulk and have low self-esteem. However, they are very attentive to their work. Equipment and facilities are needed that will help people with disabilities. These include job training or improved job practices, more labor saving equipment, and maintenance of this equipment. Moreover, cabinets or shelves should be adjustable to the needs of the disabled. Spaces for people with physical disabilities to occasionally rest are needed because they have difficulties standing or walking for long periods of time. Moreover, there is an issue with ramps and elevators. Floors can be slippery, so shoes should be developed to prevent the disabled from slipping. Most people with physical disabilities like to work in their chosen position within companies in an occupation that they enjoy and can accommodate their disabilities. Others want to own a business and be self-employed. They are working to acquire sufficient capital.

The most problematic area in the workplace is the need for physical effort. People with physical disabilities have less strength, find it difficult to climb or retrieve objects from high places, or push heavy carts. Wheelchair users require a low sloped ramp and must work in an office. There may be problems with the use of tables and file cabinets.

Environmental workplace barriers consisted of inability to reach high objects, inability to lift or push heavy objects, and inability to work quickly and deal with slippery floors. For wheelchair users, there is no storage space and they have to hang their belongings on their wheelchairs. Since there is no storage space, the tables are messy and objects may fall or be misplaced.

Environmental issues exist because there are problems related to heavy lifting and incorrect use of lifting or towing equipment. There should be grasping tools or instruction in the use of hand tools. People with physical disabilities have low grip strength and finger numbness. They would like to have devices to relieve this numbness.

There were five documented accidents in the workplace. For example, objects fell due to difficulties reaching them. There was a collision with a forklift because a person did not hear the warning. Others fell down staircases, dropped objects, and placed objects at incorrect locations.

There were seven accidents when things fell and could not be retrieved. A pallet truck ran over a person's foot. A cart malfunctioned when it was pushed. Another person cut himself with a box cutter while unpacking materials.

The environment could be improved with more shelf space. Shelf and cabinet heights should be adjustable. Moreover, wheeled carts should be properly maintained. There should be training on equipment use and its operation. Office furniture and cabinets are located too high. People with physical disabilities require labor saving devices, adjustable storage spaces, and improved access to stairs. They also think that there should be a ramp to elevators. At present, the elevators are often located far from the office. The physically disabled cannot do work that requires heavy lifting and exertion because they do not have physical strength.

A summary of interviews and observations revealed that the visually impaired needed space with trainers in places such as department stores, retail and wholesale companies (see Figure 1: the researcher collected data in Phase 1 using questionnaires, observations, and interviews with three groups of disabled people in major retail and wholesale companies in Bangkok). The visually impaired preferred to work at a main office or act as a representative of a company rather than working at a branch office due to the lack of the facilities provided for the visually impaired at the smaller branch offices. The limitations of the visually impaired restrict them to functions such as telephone operators, where they answer questions and collect information.

Environmental work barriers are mitigated by features such as wall guards, pull down doors, table corner edge protectors, and electronic equipment designed to be used by people with visual impairments.

Environmental aids for the visually impaired include contrasting colors on pavement or symbols that give directions in the workplace. Moreover, there should be wall signage to give directions in large workplaces to spaces such as bathrooms and dining rooms. The steps should not be too steep, and skid guards need to be installed. Two common accidents in the workplace include electrical shocks and bumping into table corners.

There should be tactile paving for the visually impaired, direction signage, guards for electrical outlets, walls, tables, door handles, shelves, and any surfaces that need to be touched. Electrical outlets and switches are problematic. Braille computer technology for the visually impaired is helpful. Also, the visually impaired need directional signs to help prevent them from getting lost and to help them understand daily routines. Guard forms are needed to protect from injury caused by bumping or colliding with objects and door openings. Electrical appliances and office furniture that makes work easier and more convenient will be very helpful.

The disabled prefer to work in private companies since they offer greater work security. The positions that they take should be based on the needs and abilities of the individual. Having a disability is not a barrier to working. People with disabilities are often more committed to their work.

Environmental issues that should be addressed involve office furniture for wheelchair users, the visually impaired, and appropriate communication equipment for the hearing impaired.

Materials used by disabled persons must be safe and appropriated for the tasks that they perform. The visually impaired people prefer to touch materials. Surfaces that they contact must not be harmful to the touch. Materials must not be too heavy or likely to fall or become obstacles into which they may collide.

There are many problems with office furniture, based on the individual's particular type of disability. Wheelchair users are subject to the effects of seat height and table levels, distance from the computer, and height of wall cabinets that makes them inaccessible. The visually impaired have problems with tables, falling objects, inability to find objects, crashing or bumping into obstacles, electrical outlets, and handles and hooks for clothes. They also bump into open drawers and are unable to retrieve objects from high places. Addressing these issues in the work environment would improve work effectiveness and create a sense that others care about the well-being of all employees.

A recommendation is that if a company desires to help people, the physically disabled need to be able to work effectively. Work related problems of these people need to be addressed.

## 5. Discussion

For the focus group, 15 participants were selected from major retail and wholesale companies in Bangkok such as Makro, Big C, and Lotus, instructors and experts in universal design, and the disabled. They brainstormed on appropriate design for product development. It was found that people with disabilities required improvement of workplace facilities and their work environment. However, this development needed to be an example of problem solving and improvement of work efficiency according to the principles of universal design [2]. These experts identified the following problems: office furniture, tables, and cabinets as well as chairs for the physically handicapped (wheelchairs) and the visually impaired.

**Figure 2:**
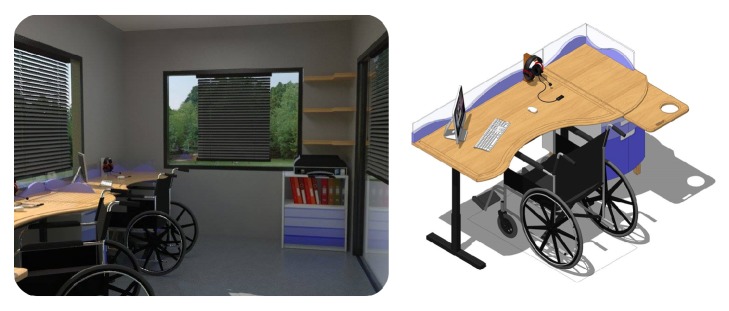
Design of a work table, side filing cabinet, and wall cabinet. The work table is adjustable, up and down. The side filing cabinet has an adjustable storage compartment and a wall cabinet that is more easily accessible [2]. Source: the current study.

**Figure 3:**
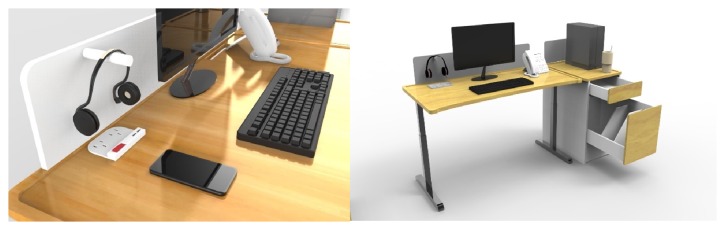
Design of guardrails and barriers, electric switches, mobile and equipment storage compartments (for canes or slates) for the visually impaired [2]. Source: the current study.

**Figure 4:**
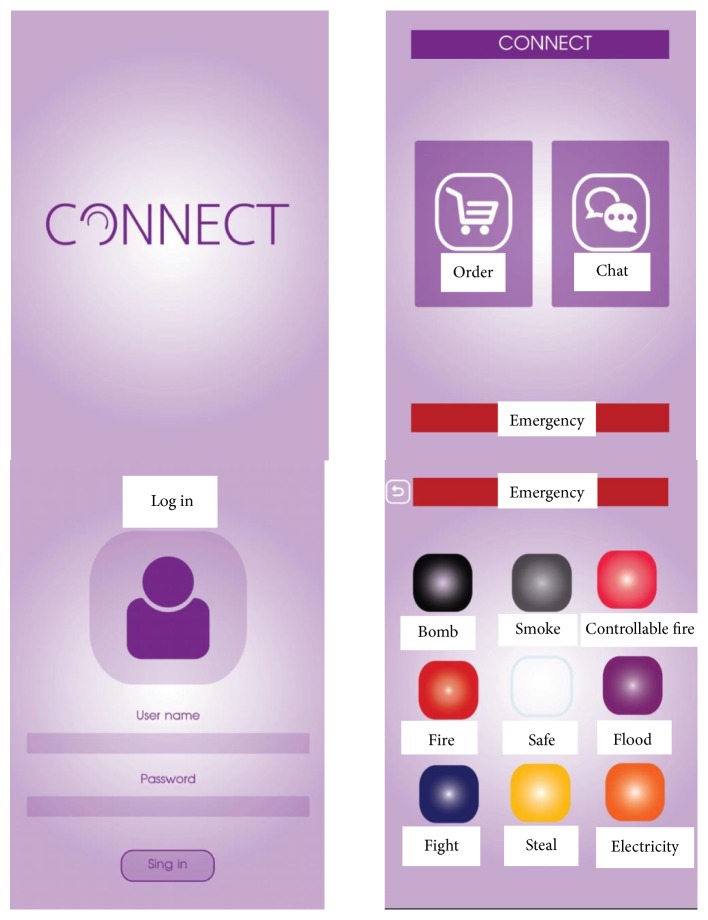
A mobile application used to assign a task to disabled staff [2]. Source: the current study.

The 15 experts summarized guidelines for problem solving as follows:

(1) For tables (see Figure 2: design of a work table, side filing cabinet, and wall cabinet), the work table is adjustable, up and down. The side filing cabinet has an adjustable storage compartment and a wall cabinet that is more easily accessible, and wheelchair users must check the wheelchair turning radius. The position of the electrical outlets should be inside tables and side cabinets. Hydraulic adjustments should be used rather than manual ones due to the low physical strength of the disabled. The price of furniture must be affordable. Recyclable material should be used to reduce the costs. Moreover, there should be guardrails and barriers to prevent collisions and injuries (see Figure 2) [10].

(2) The drawer handles of side cabinets should be recessed to prevent unwanted interaction with disabled people (see Figure 2: design of a work table, side filing cabinet, and wall cabinet). Moreover, the visually impaired have difficulties retrieving objects from the bottom of cabinets, so these should be adjustable. Table tops should have an easy-to-sign storage compartment, with signs or symbols for the visual impaired to recognize (see Figure 2).

(3) Wall cabinets are not suitable for the visual impairment due to the danger of falling of objects. There should be Braille on the outside of the cabinets to identify the contents of the cabinets. It should be of their own design (see Figure 3: design of guardrails and barriers, electric switches, mobile and equipment storage compartments (for canes or slates) for the visually impaired).

(4) A portable, inexpensive, and easy to use device should be used to give instructions to the hearing impaired. Smartphones watches or accessories used in everyday life can be suitable for this purpose. Instructions given to the hearing impaired should be easy to retrieve in the event that they are forgotten. Moreover, its use should not excessively interfere while working with a trainer (see Figure 4: a mobile application used to assign a task to disabled staff).

Development of products for the disabled must observe universal design principles. We checked that the design used these principles prior to production [11].

**Figure 5:**
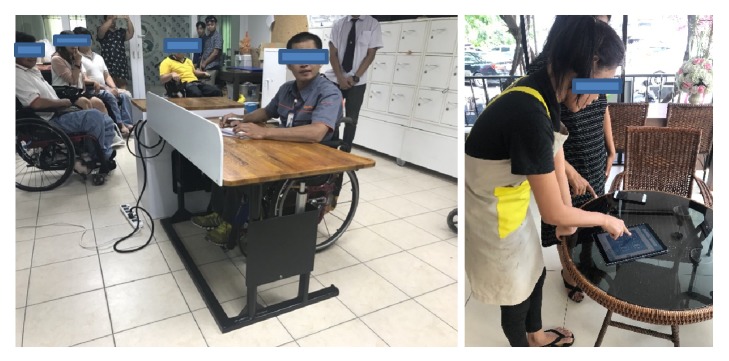
Post-use tests of the office furniture and workplace facilities (application) to improve work effectiveness of people with physical disabilities and the deaf in department stores, retail and wholesale companies for 15 people [2]. Source: the current study.

Statistical principles were used to test the effectiveness of design and development of workplace facilities and the work environments of the disabled in major retail and wholesale companies such as Makro, Big C, and Lotus. The research hypothesis was that the work effectiveness of the disabled was improved after using the developed office furniture and workplace facilities at a statistically significant level (*α* =0.05) (see Figure 5: post-use tests of the office furniture and workplace facilities (application) to improve work effectiveness of people with physical disabilities and the deaf in department stores, retail and wholesale companies for 15 people).

H_0_: *μ* for the posttest using the developed office furniture and workplace facilities ≤ *μ* for the pretest (i.e., the developments were ineffective)

H_1_: *μ* for the posttest using the developed office furniture and workplace facilities >*μ* for the pretest (i.e., the developments were effective)

Prior to analyzing the data to identify work effectiveness, the researchers checked the underlying assumption that the independent variables were normally distributed. In this study, the researchers tested this using the Kolmogorov Smirnov one-sample test and the Shapiro-Wilk test. The results revealed that the dependent variable, work effectiveness of the disabled, was normally distributed (*α* =0.05), as indicated in Table 2.

Comparison of average scores of work effectiveness of the disabled who worked at the establishments in the study showed that the average post-use test score was statistically higher than pre-use test score (*α* =0.05). This supports the research hypothesis (H_1_). The average score of work effectiveness of people with disabilities on the pretest and its standard deviation were X = 13.0667, SD = 2.93906. For the post-test, these values were X = 14.667, SD = 2.69037, as indicated in Table 3.

## 6. Conclusions and Recommendations

Visually impaired people often work in the position of telephone operators. They provide customers with information, take information, and transfer phone calls to various offices within the facility. Their most common barriers to work are problems with desks, filing cabinets, chairs, and office tools. The most common issues involve safe access to furniture without accidents. They can have problems when objects that fall off of a table cannot be retrieved. Sometimes they misplace their canes and slates and forget where they put them. They can bump into the corners of tables and have trouble finding devices. Open drawers present problems when they catch the loose clothing of the visually impaired. They cannot work with documents and files. All these problems cause problems in the work of the blind and cause problems in the workplace.

The physically disabled often work in offices. They can check goods in a warehouse to fulfill orders. Their biggest problems involve using table and file cabinets. People with disabilities have problems traveling to work and crossing the threshold of doorways. It is not easy for them to eat lunch in a cafeteria. Most of their food is kept on their wheelchairs but can accidentally fall. They have trouble using desks because they cannot get their wheelchairs under the desk. They have to move from their wheelchair to an office chair. Low mounted electoral plug is difficult to use. Objects can fall from tables and cannot be retrieved.

The hearing impaired are often chosen to restock shelves after customers have purchased materials. They work in many sections such as the clothing, food, and beverage departments as well as in warehouses. Their communications with their supervisors and coworkers are sometimes difficult leading to incorrect work orders and frequent mistakes. The deaf often cannot communicate their problems. Frustrations lead to quarrels with colleagues and resignations from their jobs.

Universal design theory is a tool that helps researchers to design systems that are safe and easy to understand, reduce the need for physical effort, and lessen fatigue and that deepen understanding about producing knowledge [5]. Equality allows everyone to work together. The ways of working can be dependent on the individual department head. A department head assigns work and when this person is changed, the deaf must learn a new way of working. The researchers of the current study developed an application to gather needed information. Written words are very useful to the hearing impaired. The video and graphics of this application were designed to be easy to understand and applicable to various departments. Data was collected about the physical workplace, desks, cabinets, and the physical view of the disabled. Things that reduce movements tend to reduce accidents such as electrical shocks. As problems are reduced, it will be easier for the disabled to work.

Filing cabinets were developed to suit the disabled and the blind. The table level was made adjustable. Electrical sockets at the table are high enough that bending is not required to plug in equipment. Storage for food and drinking water is provided and can be adjusted to have lunch with ease. Organized storage space was added.

Hearing impaired people have an application that can be used on their smartphones. This makes it easy for normal and disabled people to work together. Testing was done before and after introduction of new products. It was found that people with disabilities can work more effectively using the developed products. They experienced greater convenience and reduced accidents at work. Disabled employee had better performance and work more productively in the organization.

The disabled have many needs. There are many obstacles that have not been addressed in the current research, such as the problems of disabled people with weak hands and blind people who need to read documents.

The methodology used in this research can apply this process to the solution of other problems. For example, challenges faced by the elderly and for pregnant women need to be addressed.

This research focuses on the work of the disabled. The researchers chose to focus on department stores because they are located in every province of Thailand. Developments in the current and future studies will help disabled people throughout Thailand. Researchers can use the results of the current study to apply in future work to assist other disadvantaged groups.

This research has been recognized and endorsed by the Mahidol University Center of Ethical Reinforcement for Human Research. COA No. MU-CIRB 2018/018.1901.

## Figures and Tables

**Table 1:** Study and development of a workplace environment for people with disabilities, consulting trainers of 120 people employed at shopping malls, retail, and wholesale companies using questionnaires [2].

**Attribute**	**Number**	**Percentage**
Gender		
(i) Male	50	41.67
(ii) Female	70	58.33

Age		
(i) 20-40 years old	80	66.67
(ii) 41-60 years old	40	33.33
(iii) Over 60 years old	0	0

Education		
(i) Elementary	60	50.00
(ii) High school	15	12.50
(iii) Diploma	15	12.50
(iv) Bachelor degree	30	25.00

Working Experience		
(i) 0-1 years	63	52.49
(ii) 1-5 years	40	33.33
(iii) 5-10 years	17	14.17
(iv) Over 10 years	0	0

Position		
(i) Apparel	17	14.16
(ii) Food (transport merchandise)	15	12.50
(iii) Bakery	15	12.50
(iv) Warehouse	9	7.49
(v) Reception	15	12.50
(vi) Office Staff	15	12.50
(vii) Telephone Operator	14	11.67
(viii) HRM manager	20	16.67

Income per month (THB), with food allowance of 500 baht per month		
(i) Less than 10,000	60	50.00
(ii) 10,000-15,000	30	25.00
(iii) 15,001-20.000	10	8.33
(iv) More than 20,000	20	16.67

What is your preferred work position?		
(i) Same	96	80.00
(ii) Other (with suggestions)	24	20.00

Is the disability a barrier to work?		
(i) Yes	96	80.00
(ii) No	24	20.00

Name the areas that is barrier to work		
(i) Office Area	10	8.33
(ii) Dining Area	5	4.167
(iii) Store Area	20	16.67
(iv) Center Area	5	4.167
(v) None	80	66.67

What are the barriers to work?		
(i) Poor environment, such as no lifts, no toilets for the disabled	40	33.33
(ii) Poor facilities, no supportive equipment for workplace	80	66.67
(iii) Other causes	0	0

The problem found in the workplace environment		
(i) Climbing up and down stairs	10	8.33
(ii) Accessible toilets	10	8.33
(iii) Footpath	0	0
(iv) Loss of direction	10	8.33
(v) Tables, chairs, cabinets and furniture not suited the disabled	30	25.00
(vi) Opening and closing doors	0	0
(vii) Buying and eating foods	10	8.33
(viii) Direction and emergency signage	10	8.33
(ix) Parking	0	0
(x) Electrical appliances	40	33.33
(xi) Other	0	0

Have you ever been on the work accident?		
(i) No	70	58.33
(ii) Yes	50	41.67

Should the company improve the environment for the disabled?		
(i) Yes	80	66.67
(ii) No	40	33.33

Name the material that should be used with the disabled		
(i) Wood	50	41.67
(ii) Metal	30	25.00
(iii) Animal Hides	5	4.167
(iv) Plastic	15	12.50
(v) Rock and concrete	5	4.167
(vi) Other	15	12.50

Suitable shapes for work are		
(i) Round	20	25.00
(ii) Square	50	41.67
(iii) Rectangle	50	41.67
(iv) Hexagon	0	0
(v) Triangle	0	0
(vi) Other	0	0

What is the most problematic piece of work furniture?		
(i) Tables	50	41.67
(ii) Chairs	30	25.00
(iii) Cabinets	20	16.67
(iv) Other	20	16.67

What is the most problematic electrical equipment?		
(i) Computer	50	41.67
(ii) Electrical outlets and switches	30	25.00
(iii) Fan	0	0
(iv) Air conditioner	0	0
(v) Copier	0	0
(vi) Scanner	20	16.67
(vii) Fax	20	16.67
(viii) Other	0	0

Should the company improve the workplace facilities for the disabled?		
(i) Yes	70	58.33
(ii) No	50	41.67

What is the most problematic office equipment?		
(i) Stapler	30	25.00
(ii) Cutter	50	41.67
(iii) Punch	15	12.50
(iv) Paper cutter	5	4.167
(v) Stationery, pencil and pens	15	12.50
(vi) Paper clips	5	4.167
(vii) Other	0	0

Should the company improve the tools, equipment and machinery for the disabled?		
(i) Yes	60	50.00
(ii) No	60	50.00

Will the improvement of workplace facilities for the disabled create work effectiveness?		
(i) Yes	80	66.67
(ii) No	40	33.33

Source: the current study.

**Table 2:** Distribution test of the variable [2].

Independent Variable Group	Kolmogorov Smirnov			Shapiro-Wilk		
	Statistic	df	sig	Statistic	df	sig
Work effectiveness of people with physical disabilities						
Test	.185	15	.178	.912	15	.145
Control	.216	15	.058	.952	15	.555

(*α*: 0.05)

Source: the current study.

**Table 3:** Comparison of pre-use and post-use tests of the office furniture and workplace facilities to improve work effectiveness of people with physical disabilities in department stores, retail and wholesale companies for 15 people. A composite score was given by the users based on safety, basis in universal design, usability, and suitability for the workplace (0-5 scores were assigned for each category with a total possible score of 20) [2].

Independent Variable	$\;\overset{-}{X}$	SD	d	Std.	t	df	Sig. (2-tailed)
Pre-use testing	13.0667	2.93906	-1.600	1.76473	-3.511	14	0.003
Post-use testing	14.667	2.69037					

(*α*: 0.05)

Source: the current study.

## Data Availability

Unavailable data cannot be released because the present study is in the process. However, the study will be completely done and published online in e-Theses of the library of King Mongkut's Institute of Technology Ladkrabang in January 2019.
